# Structure of CYRI-B (FAM49B), a key regulator of cellular actin assembly

**DOI:** 10.1107/S2059798320010906

**Published:** 2020-09-23

**Authors:** Elise Kaplan, Rachael Stone, Peter J. Hume, Nicholas P. Greene, Vassilis Koronakis

**Affiliations:** a Department of Pathology, University of Cambridge, Tennis Court Road, Cambridge CB2 1QP, United Kingdom

**Keywords:** CYRI-B, FAM49B, actin assembly, cell-motility regulator, SAD, crystal structure

## Abstract

In eukaryotes, numerous fundamental processes are controlled by the WAVE regulatory complex (WRC) that regulates cellular actin polymerization, crucial for cell motility, cell–cell adhesion and epithelial differentiation. Actin assembly is triggered by interaction of the small GTPase Rac1 with CYFIP1, a key component of the WRC. Previously known as FAM49B, CYRI-B is a protein that is highly conserved across the Eukaryota and has recently been revealed to be a key regulator of Rac1 activity. Mutation of CYRI-B or alteration of its expression therefore leads to altered actin nucleation dynamics, with impacts on lamellipodia formation, cell migration and infection by intracellular pathogens. In addition, knockdown of CYRI-B expression in cancer cell lines results in accelerated cell proliferation and invasiveness. Here, the structure of *Rhincodon typus* (whale shark) CYRI-B is presented, which is the first to be reported of any CYRI family member. Solved by X-ray crystallography, the structure reveals that CYRI-B comprises three distinct α-helical subdomains and is highly structurally related to a conserved domain present in CYFIP proteins. The work presented here establishes a template towards a better understanding of CYRI-B biological function.

## Abstract 2

The crystal structure of CYRI-B is revealed, providing a template to understand the role of this highly conserved eukaryotic protein in a variety of actin-dependent cellular processes.

## 1. Introduction   

Actin filament dynamics are central to a myriad of essential cellular processes such as cell migration, division and intracellular trafficking (Pollard & Cooper, 2009 ▸; Rottner *et al.*, 2017 ▸; Rottner & Schaks, 2019 ▸). Actin assembly is nucleated by cellular machines such as the ubiquitous Arp2/3 complex, which drives the generation of the branched actin networks underlying processes including lamellipodia formation and cell motility (Buracco *et al.*, 2019 ▸). Arp2/3 activity is regulated by nucleation-promoting factors (NPFs), which thus provide spatial and temporal control of these processes (Campellone & Welch, 2010 ▸; Rotty *et al.*, 2013 ▸). The best-characterized NPFs are those belonging to the Wiskott–Aldrich syndrome protein (WASP) family, which stimulate Arp2/3 via C-terminal VCA (verprolin-homology, central and acidic regions) domains (Pollitt & Insall, 2009 ▸; Alekhina *et al.*, 2017 ▸). The WASP family members WAVE1, WAVE2 and WAVE3 are central to cell motility and protrusion formation (Kurisu & Takenawa, 2009 ▸; Rottner & Schaks, 2019 ▸), and each functions as part of a heteropentameric complex termed the WAVE regulatory complex (WRC; Chen *et al.*, 2010 ▸, 2014 ▸). In the resting state, the WAVE VCA domain is hidden in the WRC structure (Ismail *et al.*, 2009 ▸; Chen *et al.*, 2010 ▸). Multiple signals have been reported that can trigger exposure of the WAVE VCA domain and consequent activation of Arp2/3, the best-characterized of these being interaction with the small GTPase Rac1 (Miki *et al.*, 1998 ▸; Ismail *et al.*, 2009 ▸; Chen *et al.*, 2010 ▸), which is crucial for cell migration (Steffen *et al.*, 2013 ▸). There are two possible sites of Rac1 interaction on the WRC. The original ‘A’ (‘adjacent’) site is located in the N-terminal part of CYFIP1 (Chen *et al.*, 2010 ▸), which is a key component of the WRC, while the newly identified ‘D’ (‘distant’) site is situated at the C-terminal extremity of the protein (Chen *et al.*, 2017 ▸). While two Rac1 molecules can simultaneously bind to the WRC *in vitro* (Chen *et al.*, 2017 ▸), only the ‘A’ site was found to be essential for its activation *in vivo* (Schaks *et al.*, 2018 ▸). Several studies reported that like CYFIP1, FAM49B, a well conserved eukaryotic protein, interacts with the active form of Rac1 (Shang *et al.*, 2018 ▸; Fort *et al.*, 2018 ▸). FAM49B was consequently renamed CYRI-B for CYFIP-related Rac1 interactor (Fort *et al.*, 2018 ▸). CYRI-B binding has been reported to block various Rac1-dependent signalling cascades in the cell, thereby controlling multiple critical cellular functions including T-cell activation (Shang *et al.*, 2018 ▸), membrane protrusion, chemotaxis and cell migration (Fort *et al.*, 2018 ▸; Whitelaw *et al.*, 2019 ▸). By negatively regulating Rac1 signalling, CYRI-B also reduces the entry of several intracellular bacterial pathogens into both phagocytic and non-phagocytic host cells, and indeed is targeted for ubiquitin-mediated destruction by the action of an injected *Salmonella* virulence protein (Yuki *et al.*, 2019 ▸). CYRI-B has also been suggested to play a role in certain cancers. Thus, the expression of CYRI-B in pancreatic ductal adenocarcinoma cells is downregulated in the tumour microenvironment, leading to increased cell proliferation and invasion (Chattaragada *et al.*, 2018 ▸). Furthermore, knockdown of CYRI-B expression increases cell proliferation in colorectal and liver cancer cell lines (Long *et al.*, 2019 ▸), suggesting that CYRI-B may act as a tumour suppressor.

Here, we present the structure of *Rhincodon typus* (whale shark) CYRI-B, which is the first structure of any member of the CYRI protein family. Solved by X-ray crystallography at 2.4 Å resolution using the SAD method, the structure reveals three distinct, entirely α-helical, subdomains and high structural similarity to a conserved domain of CYFIP1. Our work provides a basis to better understand the diverse and important roles of the CYRI protein family in eukaryotic cells.

## 2. Materials and methods   

### 2.1. Cloning, expression and purification of CYRI-B (FAM49B)   

DNA fragments encoding CYRI-B (FAM49B) from *Anthurium amnicola*, *Bison bison bison*, *Crocodylus porosus*, *Homo sapiens*, *Lingula anatina*, *Ornithorhynchus anatinus*, *Orussus abietinus*, *Rhincodon typus*,* Tetranycus urticae* and *Tupaia chinensis* were synthesized (IDT) and cloned into pET-11a using Gibson assembly (Gibson *et al.*, 2009 ▸). The fragments were designed to include an N-terminal solubility-enhancement tag (SET2, Stratagene), a hexahistidine tag (His tag) and a Tobacco etch virus (TEV) protease cleavage site upstream of the CYRI-B coding sequence.

*Escherichia coli* Rosetta (DE3) cells (Novagen) bearing the appropriate expression plasmid were grown at 37°C in 2×YT medium supplemented with 50 µg ml^−1^ ampicillin and 20 µg ml^−1^ chloramphenicol. When the cells reached an OD_600_ of 0.8, the temperature was reduced to 18°C and protein expression was induced with 0.1 m*M* IPTG. After 16 h at 18°C, the bacteria were harvested by centrifugation at 6000*g* for 10 min at 4°C and the pellet was resuspended in 40 ml lysis buffer composed of 20 m*M* Tris pH 7.4, 150 m*M* NaCl, 0.25 m*M* Bond-Breaker TCEP Solution (Thermo Scientific) and one tablet of EDTA-free protease-inhibitor cocktail (Roche). The bacteria were lysed by cell disruption (Constant Systems) at 207 MPa and the lysate was clarified by centrifugation for 1 h at 60 000*g* and 4°C. The clarified lysate was then incubated with 2 ml Ni–NTA agarose (Qiagen) for 1 h at 4°C. The resin was washed with 20 ml wash buffer (20 m*M* Tris pH 7.4, 500 m*M* NaCl, 0.25 m*M* TCEP) supplemented with 10 m*M* imidazole before elution with the same buffer containing only 150 m*M* NaCl and supplemented with 250 m*M* imidazole. The eluate was dialyzed against 5 l dialysis buffer (20 m*M* Tris pH 7.4, 150 m*M* NaCl, 5 m*M* β-mercaptoethanol). The N-terminal His tag was removed using His-tagged TEV protease produced from plasmid pTH24:TEVSH as described in van den Berg *et al.* (2006 ▸). Cleavage reactions utilized 250 µ*M* CYRI-B and 185 µ*M* TEV protease in a total volume of 10 ml and were incubated for 4 h at 22°C. Free His tags, uncleaved CYRI-B protein and TEV protease were removed by several passages over 2 ml nickel resin (Bio-Rad Profinity). Recombinant CYRI-B was concentrated to 10 mg ml^−1^ using a 10 kDa molecular-weight cutoff Amicon ultra centrifugal filter (Millipore), supplemented with 10% glycerol and stored at −80°C.

For preparation of selenomethionine (SeMet)-derivatized CYRI-B, the metabolic inhibition protocol was used (Van Duyne *et al.*, 1993 ▸). *E. coli* Rosetta (DE3) cells carrying the CYRI-B expression plasmid were grown in M9 minimal medium supplemented with 50 µg ml^−1^ carbenicillin, 0.2% glucose, 2 m*M* MgSO_4_, 0.1 m*M* CaCl_2_ and 0.001% thiamine to an OD_600_ of 0.8. At this point, the culture was supplemented with 60 mg ml^−1^ SeMet, 50 mg ml^−1^ leucine, isoleucine and valine and 100 mg ml^−1^ lysine, phenylalanine and threonine. The temperature was reduced to 18°C and the cells were grown for a further 15 min before the induction of protein expression with 0.1 m*M* IPTG. After a further 16 h, the cells were pelleted by centrifugation at 6000*g* for 6 min. The SeMet-labelled CYRI-B was then purified and the His tag was removed by TEV proteolysis as described for the native protein.

### 2.2. Analytical size-exclusion chromatography   

Size-exclusion chromatography (SEC) was utilized to confirm the oligomeric state of the native and SeMet-derivatized CYRI-B proteins. SEC was performed at room temperature using an ÄKTA pure FPLC system (GE Healthcare) equipped with a Superdex 75 10/300 GL column in 20 m*M* Tris pH 7.5, 150 m*M* NaCl, 5 m*M* β-mercapto­ethanol. A total of 0.5 mg native protein or 0.3 mg of the less soluble SeMet derivative was applied onto the column in a final volume of 100 µl.

### 2.3. Native and SDS–PAGE gels   

The purity and oligomeric state of the recombinant CYRI-B proteins were analyzed using 7% native and 10% SDS–PAGE under non-reducing and reducing conditions, respectively. Protein bands were visualized using Instant Blue (Expedeon).

### 2.4. Crystallization   

Crystallization trials were set up in 2-Drop MRC plates (Molecular Dimensions) using a Mosquito robot (TTP Labtech). Crystals were grown using the sitting-drop vapour-diffusion method. Initial screening was performed using the commercial crystallization screens Structure Screen, MIDAS*plus*, JCSG-*plus* (Molecular Dimensions) and SaltRx (Hampton Research). Subsequent optimization utilized homemade screens.

Native CYRI-B protein at 10 mg ml^−1^ was mixed with the reservoir solution in a 1:2 ratio in a final volume of 1 µl over a reservoir of 80 µl. Crystals were obtained at 15°C in 0.2 *M* ammonium acetate, 0.1 *M* MES pH 6.5, 30%(*w*/*v*) glycerol ethoxylate using crystals obtained in 0.1 *M* MES pH 6.5, 7–10% PEG 8000, 20% PEG 1000 as seeds. Single orthorhombic prisms of approximately 300 × 40 × 15 µm in size appeared the following day. Crystals were cryoprotected by the reservoir liquor and cooled in liquid nitrogen.

The SeMet-substituted CYRI-B was less soluble than the native protein and was consequently only concentrated to 2.6 mg ml^−1^ in a buffer consisting of 20 m*M* Tris pH 7.5, 150 m*M* NaCl, 5 m*M* β-mercaptoethanol. Crystallization was carried out by mixing the protein with a precipitant solution composed of 0.2 *M* ammonium acetate, 0.1 *M* bis-Tris pH 5.5, 25% PEG 3350 in a 1:2 ratio in a final volume of 1 µl. Crystals were obtained after one day with the same seed stock as used for the native protein but were slightly smaller (approximate size 50 × 20 × 10 µm). They were cryoprotected with the reservoir solution supplemented with 10%(*v*/*v*) glycerol. Images of protein crystals together with a typical diffraction pattern are shown in Supplementary Figs. S1(*a*) and S1(*b*).

### 2.5. Data collection and structure determination   

A single-wavelength anomalous diffraction (SAD) data set was collected for SeMet-labelled CYRI-B on beamline I03 at Diamond Light Source (DLS). A total of 720 images (0.04 s exposure, 0.5° oscillation) were collected on a PILATUS3 6M detector from a single crystal by illumination at 12 900 eV (0.9611 Å). The data were indexed and integrated with *iMosflm* (Battye *et al.*, 2011 ▸) in space group *P*2_1_2_1_2_1_ and scaled with *AIMLESS* (Evans & Murshudov, 2013 ▸) to a maximum resolution of 2.50 Å. The last 120 images were excluded owing to radiation damage, as judged from the batch *I*/σ(*I*) and *R*_merge_ statistics. Anomalous scatterers were located with the *HySS* (*Hybrid Substructure Search*; Grosse-Kunstleve & Adams, 2003 ▸) module of *Phenix* (Adams *et al.*, 2010 ▸; Liebschner *et al.*, 2019 ▸). The 15 heavy-atom sites were input into *Phaser* (McCoy *et al.*, 2007 ▸) for SAD phasing followed by density modification with *Parrot* (Cowtan, 2010 ▸) and model building with *Buccaneer* (Cowtan, 2006 ▸). The overall figure of merit for the final SAD phases was 0.48 and increased to 0.57 after the first density modification. After each refinement step with *REFMAC* (Murshudov *et al.*, 2011 ▸), model inspection and building were performed with *Coot* (Emsley *et al.*, 2010 ▸). The data were finally reprocessed at 2.40 Å resolution and the model was refined with cycles of manual modification with *Coot* and refinement first with *REFMAC* and then with *Phenix* for the last cycles, including two TLS groups and automatic X-ray/stereochemistry weights. The final model has *R*_work_ and *R*_free_ factors of 0.2048 and 0.2415, respectively.

The native data set was collected on beamline I04 at DLS. 720 images (0.05 s exposure, 0.5° oscillation) were collected on a PILATUS 6M-F detector. The data were processed with *iMosflm* in space group *P*2_1_2_1_2_1_ and scaled with *AIMLESS*, giving a data set composed of 13 266 unique reflections to a maximum of 2.37 Å resolution. The structure was solved by molecular replacement with *Phaser* using the model obtained from the SeMet-derivatized data set. The model was then improved by several rounds of manual building with *Coot* and refinement first with *REFMAC* and then with *Phenix* for the last cycles, using two TLS groups and automatic X-ray/stereochemistry constraints. The final model has *R*_work_ and *R*_free_ factors of 0.2251 and 0.2772, respectively.

During refinement, eight ordered water molecules were manually added to both the SeMet-derivatized and the native structures. In both data sets, the electron density was too weak to build residues 171–175. Both models were validated using tools from *Coot*, *RAMPAGE* (Lovell *et al.*, 2003 ▸) and *PROCHECK* (Laskowski *et al.*, 1993 ▸). Data-collection and structure-refinement statistics are listed in Table 1 ▸. The coordinates and structure factors of the native and SeMet-derivatized proteins have been deposited in the Protein Data Bank (PDB) with accession codes 6yjk and 6yjj, respectively.

### 2.6. Electrostatic potential calculations and figure preparation   

Electrostatic potential profiles were calculated and visualized in *PyMOL* using the *APBS* plugin (version 2.1; Baker *et al.*, 2001 ▸; Jurrus *et al.*, 2018 ▸) and PQR files generated by the *PDB*2*PQR* server (Dolinsky *et al.*, 2004 ▸, 2007 ▸). The surface potential was set to ±4.0 *kT* e^−1^. All figures were generated with *PyMOL* version 2.2.2.

## 3. Results and discussion   

### 3.1. Structure of CYRI-B (FAM49B), an α-helical protein   

To facilitate structural studies, we cloned and expressed CYRI-B (FAM49B) from *H. sapiens* and nine homologues. Most of the proteins either did not express well (*O. anatinus* and *T. chinensis*), were unstable after purification (*A. amnicola*, *B. bison bison* and *O. abietinus*) or failed, so far, to crystallize (*L. anatina*, *C. porosus*, *T. urticae* and *H. sapiens*). However, we successfully produced crystals of whale shark (*R. typus)* CYRI-B. After optimization and screening of multiple crystals, we were able to obtain diffraction data at 2.37 Å resolution. We attempted to solve the structure by molecular replacement using the DUF1394 domain of CYFIP1 (PDB entry 3p8c; Chen *et al.*, 2010 ▸), as it shares 21% sequence identity with whale shark CYRI-B and is predicted to be structurally similar (Yuki *et al.*, 2019 ▸). However, these efforts were unsuccessful.

We therefore produced SeMet-substituted protein, which allowed us to determine the structure of CYRI-B by SAD phasing. The native and SeMet-derivatized protein crystals have similar unit-cell dimensions and belong to the same ortho­rhombic space group, *P*2_1_2_1_2_1_. They were solved at resolutions of 2.37 and 2.40 Å, respectively. Data-collection and refinement statistics are given in Table 1 ▸ and represen­tative electron densities for the native and SeMet-substituted structures are shown in Supplementary Movies S1 and S2, respectively. In both cases, a single molecule is present in the asymmetric unit. The two models are nearly identical and could be aligned with a C^α^ r.m.s.d. of 0.29 Å for 320 atoms.

**Figure 1:**
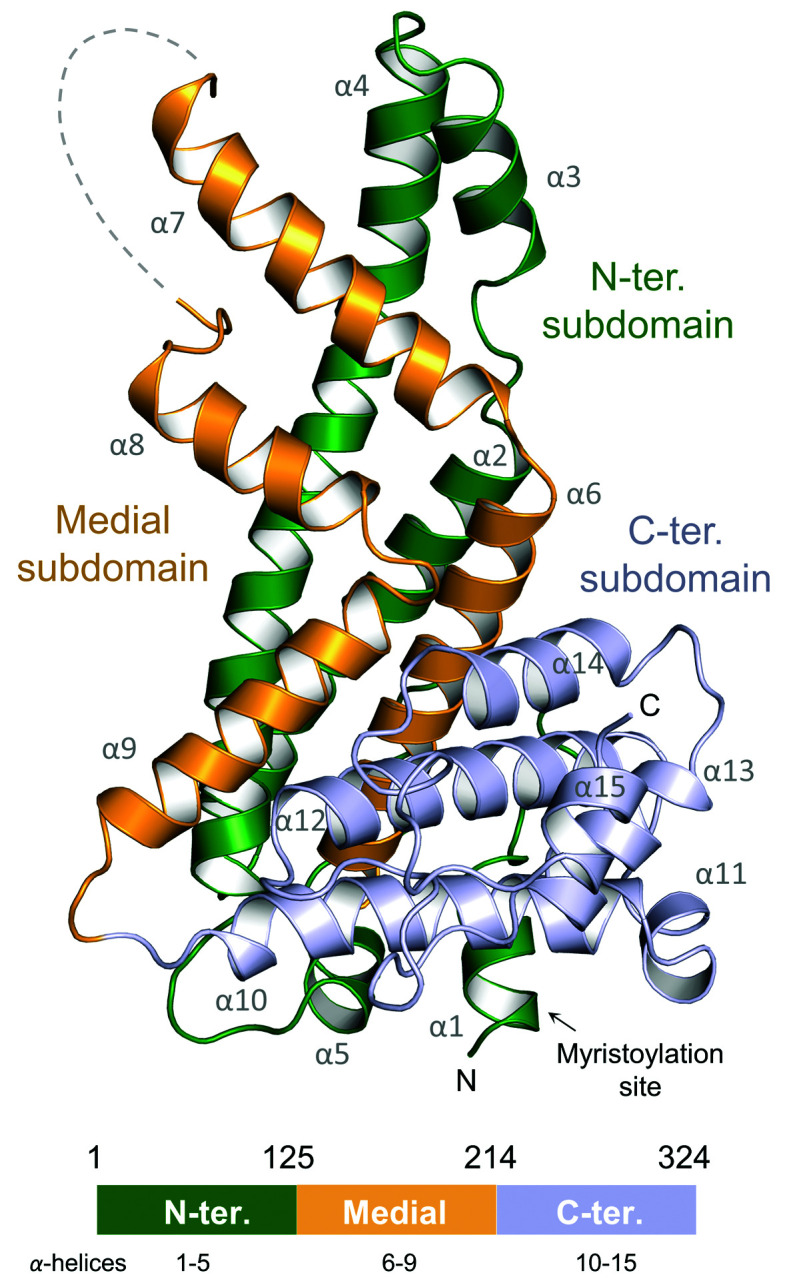
Crystal structure of CYRI-B. Cartoon representation of native CYRI-B from *R. typus* showing the N-terminal (green), medial (yellow) and C-­terminal (blue) subdomains. The myristoylation site of the protein (Fort *et al.*, 2018 ▸) is indicated. A schematic linear organization of the protein subdomains is represented underneath.

The structure of CYRI-B (FAM49B) is shown in Fig. 1 ▸. It reveals a protein comprised solely of α-helices which can be divided into three linear subdomains. The N-terminal sub­domain extends to residue 125 and consists of five α-helices, three of which form a 60 Å elongated α-helical hairpin that runs the entire length of the protein. The medial sub­domain extends from residues 126 to 214 and exhibits a 90° broken antiparallel helical hairpin that covers one face of the N-terminal subdomain. The two antiparallel helices α6 and α9 split at the lowest part to surround the extended α4 helix of the N-terminal subdomain. Finally, the C-terminal subdomain (residues 215–324) consists of six α-helices which associate into a globular bundle located at the base of the structure. This bundle caps the lower half of the medial subdomain and is elevated above helices α1 and α5 of the N-terminal sub­domain.

In both the native and the SeMet-derivatized structures, the flexible loop composed of residues 171–175 was too disordered to build and is therefore missing from the final models. This loop connects helices α7 and α8 of the medial subdomain. A non-native serine, residual from the purification strategy, is present at the N-terminus of the structure.

CYRI-B possesses five cysteines: one is located in the N-terminal region, while the C-terminal subdomain has four. In the C-terminal region, the two cysteines Cys231 and Cys253 from helices α10 and α12 are located too far apart (>3 Å) to form an internal disulfide bond in our structure (Supplementary Fig. S2*a*). However, it is likely that under non-reducing conditions these two helices are stabilized by a disulfide. Gly2, which is *N*-myristoylated *in vivo* (Fort *et al.*, 2018 ▸), is located at the base of the structure (Fig. 1 ▸) and is freely accessible.

**Figure 2:**
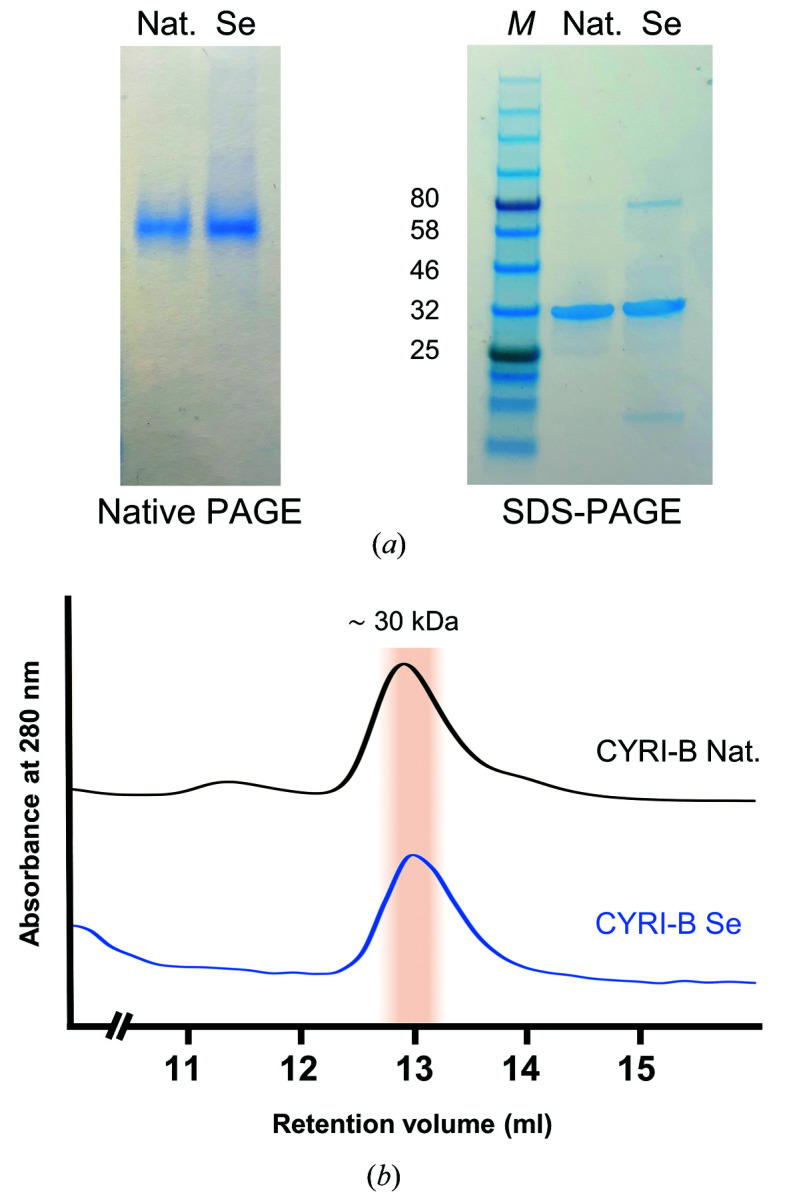
Oligomeric state of CYRI-B. (*a*) Native PAGE (left) and SDS–PAGE (right) gels of native (Nat.) and SeMet-derivatized (Se) CYRI-B proteins. Molecular masses of protein standards (lane *M*) are shown in kDa. (*b*) Size-exclusion chromatography profiles of the indicated proteins.

Biochemical analyses of *R. typus* CYRI-B were conducted to confirm the oligomeric state of the protein found by X-ray crystallography. As presented in Fig. 2 ▸(*a*), the native and SeMet-derivative proteins migrate as a single band on both native and SDS–PAGE gels. Under denaturing conditions, the proteins migrate with an apparent molecular mass of ∼30 kDa, which is close to the theoretical value of 37 kDa for CYRI-B. Further analysis of the purified proteins by size-exclusion chromatography (SEC) revealed a single peak which elutes close to 30 kDa on a calibrated gel-filtration column (Fig. 2 ▸ *b*).

The gel electrophoresis and SEC results corroborate the crystallographic data, confirming the monomeric state of CYRI-B in solution.

### 3.2. Comparison with human CYRI-B and relatives   

#### 3.2.1. Whale shark and human CYRI-B are almost indistinguishable   

**Figure 3:**
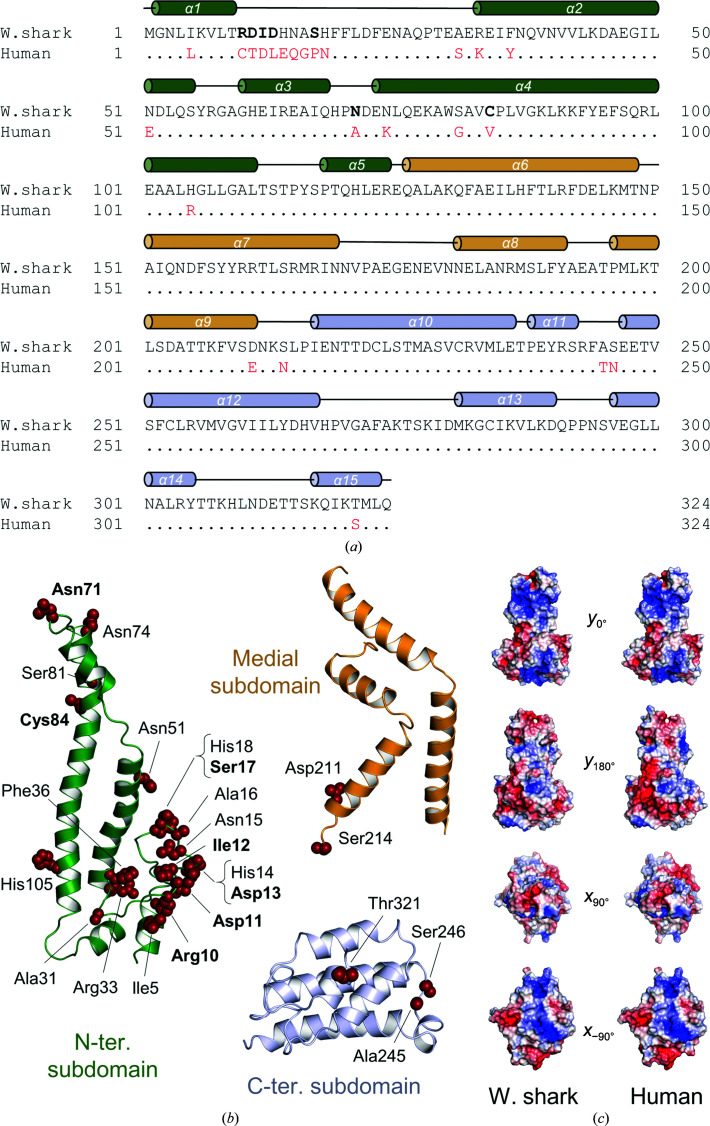
Comparison of human and whale shark CYRI-B reveals high sequence similarity. (*a*) Alignment of whale shark and human protein sequences, where divergent amino acids are displayed in red and identical residues are represented by dots. The secondary structure of whale shark CYRI-B is shown above the sequence using the same colour code as in Fig. 1 ▸. The alignment was generated with the NCBI *BLAST* program (Altschul *et al.*, 1997 ▸, 2005 ▸). (*b*) Side chains of residues that are dissimilar between the whale shark and human proteins are represented as spheres on each subdomain of the whale shark CYRI-B structure. Non-conservative differences between the two proteins are shown in bold in (*a*) and (*b*). (*c*) Views of the electrostatic surface of whale shark (left) and human (*Phyre* homology model; right) CYRI-B proteins. A 360° rotation tour is presented in Supplementary Movie S3.

CYRI proteins are ubiquitous and highly conserved, as illustrated by the alignment of sequences from multiple species (Supplementary Fig. S3*a*). For example, whale shark and human CYRI-B share a sequence identity of 93% (Supplementary Fig. S3*b*). Thus, over the 324 residues of both proteins, 12 substitutions are conservative, five are semi-conservative and only seven lead to a modification of the amino-acid physico-chemical properties (Fig. 3 ▸ *a*). Most of these substitutions localize to residues 10–18, which are located in a loop connecting helices α1 and α2 within the N-terminal subdomain (Fig. 3 ▸ *b*). This region is poorly conserved in CYRI-B proteins (Supplementary Fig. S3*a*) and is outside the presumed Rac1-binding region (Fort *et al.*, 2018 ▸). A few other mutations are located at the apex of this sub­domain. The medial and C-terminal subdomains are almost entirely conserved between the two species, containing only two and three conservative substitutions, respectively. As a result, the surface-charge distributions of whale shark CYRI-B and the human homology model are near-identical (Fig. 3 ▸ *c* and Supplementary Movie S3).

#### 3.2.2. Comparison of CYRI-A and CYRI-B isoforms   

Two isoforms of CYRI proteins exist in many species: CYRI-A and CYRI-B. Some cells express only CYRI-B, such as Jurkat T cells, while others express both (mouse T cells) (Shang *et al.*, 2018 ▸). The two proteins have a sequence identity of approximately 80% across multiple species, including whale shark and human (Supplementary Fig. S3*b*), yet are functionally distinct. While a multitude of roles have been ascribed to CYRI-B, a clear function for CYRI-A has not been defined.

**Figure 4:**
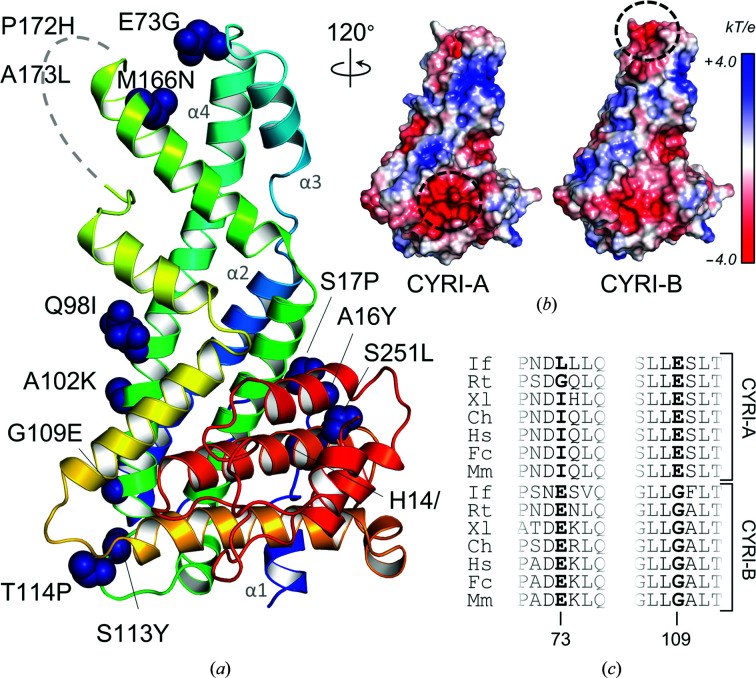
Comparison of whale shark CYRI-A and CYRI-B. (*a*) Radical substitutions between CYRI-A and CYRI-B as indicated by *Clustal Omega* (Sievers *et al.*, 2011 ▸) are shown as blue spheres on the CYRI-B structure. The corresponding residues in CYRI-B are indicated. (*b*) Electrostatic surfaces of CYRI-A (*Phyre* homology model) and CYRI-B (crystal structure). (*c*) Sequence alignment of multiple CYRI-A and CYRI-B proteins highlighting residues 73 and 109 (*R. typus* CYRI-B numbering). Abbreviations are as follows: If, *Ictalurus furcatus*; Rt, *R. typus*; Xl, *Xenopus laevis*; Ch, *Crotalus horridus*; Hs, *H. sapiens*; Fc, *Felis catus*; Ms, *Mus musculus*. The full sequence alignment is provided in Supplementary Fig. S3(*a*).

We compared the two isoforms of the whale shark protein and mapped the 13 substitutions which lead to modification of the amino-acid physico-chemical properties onto our CYRI-B structure (Fig. 4 ▸ *a*). Most of them are located in the N-terminal subdomain, some on the flexible loop located between helices α1 and α2 and several along the extended α4 helix. Some of these substitutions modify the surface electrostatic potential (Fig. 4 ▸ *b*). Thus, CYRI-A is predicted to have a negatively charged patch at the bottom of the structure, where Gly109 in the CYRI-B isoform is replaced by a glutamate in the CYRI-A protein. Conversely, the presence of a glutamate (Glu73), absent in CYRI-A, leads to the formation of a negative patch at the apex of CYRI-B. Sequence analysis reveals that these differences are conserved in CYRI proteins from other species (Fig. 4 ▸ *c*), but until more is known about the function of CYRI-A, the significance of these differences remains unclear.

### 3.3. Similarity to CYFIP1 from the WAVE regulatory complex (WRC)   

**Figure 5:**
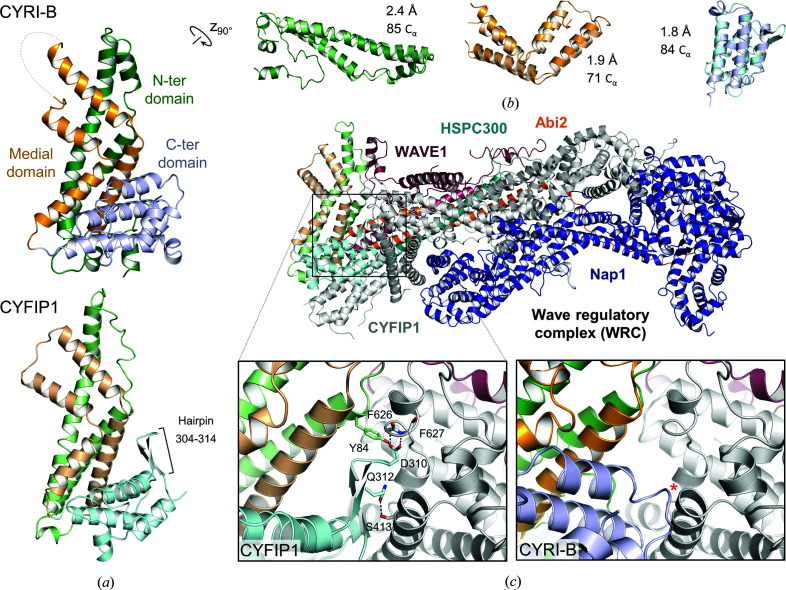
CYRI-B and CYFIP1 present a high degree of structural similarity. (*a*) Side-by-side view of the CYRI-B (left) and CYFIP1 DUF1394 domain (PDB entry 3p8c; right) structures. The colour code of CYRI-B is as in Fig. 1 ▸. The presence of an extra hairpin in CYFIP1 is indicated. (*b*) Secondary-structure alignment of the N-terminal (green), medial (yellow) and C-terminal (blue) subdomains of the two proteins. The r.m.s.d. value and number of C^α^ atoms are shown beside each alignment (from *SUPERPOSE*; Krissinel & Henrick, 2004 ▸). (*c*) Top, overall structure of the WAVE regulatory complex (WRC; PDB entry 3p8c). The DUF1394 domain of CYFIP1 is coloured as in (*a*). Bottom left, close-up view of the 304–314 hairpin of CYFIP1 showing residues involved in hydrogen-bond interactions with the rest of the protein (grey). Bottom right, superposition of CYRI-B on the DUF1394 domain of CYFIP1 showing the absence of the 304–314 hairpin. An asterisk indicates a steric clash.

The ubiquitous CYFIP protein is a component of the WAVE regulatory complex (WRC). Two CYFIP isoforms exist, CYFIP1 and CYFIP2, which both contain a DUF1394 domain (residues 59–301; human numbering) that is also shared by CYRI proteins. Despite a relatively low sequence identity of 21% between human CYRI-B and the human CYFIP1 DUF1394 domain, the two regions are predicted to be structurally homologous (Yuki *et al.*, 2019 ▸). Comparison of the CYFIP1 DUF1394 domain (PDB entry 3p8c) with our structure of CYRI-B confirms this prediction (Fig. 5 ▸ *a*). The two proteins are strikingly similar and can be superposed with an r.m.s.d. of 3.4 Å over 240 C^α^ atoms and of between 1.8 and 2.4 Å for the individual N-terminal, medial and C-terminal subdomains (Fig. 5 ▸ *b*).

Interestingly, the disordered loop (residues 169–181) connecting α7 and α8 is six residues longer than the corresponding region of CYFIP1 (residues 195–201). This loop constitutes one of the less conserved regions in the CYRI-A and CYRI-B proteins (Supplementary Fig. S3*a*). Residues 171–175, which are missing in our structure, could not be built based on the CYFIP1 structure as the flanking regions of the loop fold slightly differently in the two proteins (Supplementary Figs. S4*a* and S4*b*). Thus, helix α7 of CYRI-B is one turn longer than the corresponding helix in CYFIP1. The supplementary turn allows an interaction between Asn74 and the side chain of Gln318 belonging to a symmetry mate. Similarly, helix α8 of CYRI-B terminates one turn earlier, with the loop unfolding towards the back of the structure, where Asn177 interacts with the main chain of Asp290 belonging to a different symmetry mate. Moreover, the position of the loop in CYFIP1 is not compatible with the packing of the CYRI-B crystal (Supplementary Fig. S4*c*). Indeed, the α4 apex of a symmetry mate would clash with an extra α-turn of α8, whereas the packing of the WRC crystal (PDB entry 3p8c) allows both conformations of this linker region.

We examined the conservation of the spatially proximal cysteines Cys231 and Cys253 (see Section 3.1) in CYRI proteins and their CYFIP homologues. These cysteines are conserved in both the CYRI-A and CYRI-B isoforms, but in the CYFIP homologues, only the equivalent of Cys231 is conserved (Supplementary Fig. S2*b*). Examination of CYFIP protein sequences and the CYFIP1 structure (PDB entry 3p8c; Chen *et al.*, 2010 ▸) does not reveal a plausible partner for Cys231. The significance of these conserved cysteines for the biological function of CYRI and CYFIP remains to be clari­fied.

The comparison of the two protein structures also highlights differences in the C-terminal subdomain. In CYRI-B, a loop connects α13 and α14, whereas in the equivalent region of CYFIP1, an 11-residue β-hairpin is present (Fig. 5 ▸ *a*). This hairpin mediates both intradomain and interdomain inter­actions in CYFIP1 (Fig. 5 ▸ *c*, bottom left). Thus, the side chain of Asp310, which is located at the apex of the hairpin, forms a hydrogen bond to the Tyr84 hydroxyl of the N-terminal subdomain and the main-chain amides of Phe626 and Phe627 from the FragX-IP domain (residues 389–1222 of CYFIP1). Interaction with the FragX-IP domain is further stabilized by a hydrogen bond between Gln312 and the side chain of Ser413.

**Figure 6:**
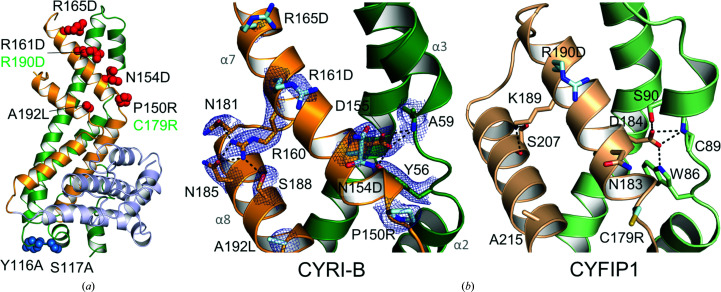
Predicted association of Rac1 with CYRI-B and CYFIP1. (*a*) Site-directed mutagenesis data mapped onto the *R. typus* CYRI-B crystal structure. Mutations in CYRI-B (black labels) that impair (red spheres) or do not affect (blue spheres) its function *in vivo* (Shang *et al.*, 2018 ▸) are shown. Green labels indicate mutations in the CYFIP1 DUF1394 domain that perturb the association of the WRC complex with Rac1 (Chen *et al.*, 2010 ▸). (*b*) Close-up views of the predicted Rac1-interacting regions of CYRI-B (left) and CYFIP1 (right), coloured as in Fig. 5 ▸. Residues leading to effective mutations in (*a*) are represented as cyan sticks with the corresponding mutated residue indicated. Electron densities of CYRI-B side chains are shown as a blue mesh contoured at a σ level of 1.

CYFIP1 has been shown to bind Rac1 (Chen *et al.*, 2010 ▸), and mutations that impair binding form a patch on CYFIP1 (Fig. 6 ▸ *a*). Two of these mutations, C179R and R190D, are positioned on the DUF1394 domain of CYFIP1. Similarly, Shang and coworkers depicted the interaction of CYRI-B with Rac1 using co-immunoprecipitation and GST pull-down assays (Shang *et al.*, 2018 ▸). The association was reinforced with active forms of Rac1, *i.e.* the nucleotide-bound form and the constitutively activated G12V mutant. They subsequently identified a series of mutations which abolished the function of CYRI-B *in vivo*.

We mapped these mutations onto our structure of CYRI-B (Figs. 6 ▸ *a* and 6 ▸ *b*). None of the residues are involved in intraprotein interactions, which is also the case for the two CYFIP1 mutants described to diminish the interaction between a modified version of the WRC and Rac1 (Fig. 6 ▸ *b*; Chen *et al.*, 2010 ▸).

In both CYRI-B and CYFIP1, helix α7, which comprises the majority of the residues that are predicted to constitute the Rac1-binding region, is stabilized by interactions with the antiparallel helix α8 and through a conserved aspartate that interacts with the loop joining helices α2 and α3 of the N-terminal subdomain (Fig. 6 ▸ *b*, left). Thus, in CYRI-B, Asp155 forms hydrogen bonds to the hydroxyl group of Tyr56 and the backbone amide group of Ala59, while the equivalent CYFIP1 residue, Asp184, forms a hydrogen-bonding network with Trp86, Cys89 and Ser90 (Fig. 6 ▸ *b*, right).

The highly conserved Arg161 of CYRI-B adopts an identical orientation to the equivalent residue in CYFIP1 (Arg190). This arginine is believed to play a central role in the association with Rac1 (Chen *et al.*, 2010 ▸; Fort *et al.*, 2018 ▸; Shang *et al.*, 2018 ▸). The side chain of a second conserved arginine (Arg160) in CYRI proteins forms extensive hydrogen bonds to the side-chain carbonyl groups of Asn181 and Asn185 and to the hydroxyl group of Ser188, whereas the equivalent residue in CYFIP1 (Lys189) interacts with the carbonyl and hydroxyl groups of Ser207. Interestingly, mutation of the conserved Arg160 to aspartate abolishes the interaction of CYRI-B with the activated Q61L Rac1 variant (Fort *et al.*, 2018 ▸). It is possible that this is owing to a local disruption of the secondary structure as Arg160 stabilizes the interaction of α7 and α8. Alternatively, the presence of two consecutive positively charged amino acids may be crucial for the recruitment of Rac1, in which case Arg160 may alter its conformation to interact with specific residues of Rac1.

## 4. Conclusion   

In summary, we have revealed the structure of CYRI-B, also called FAM49B, the only described negative regulator of cellular actin assembly mediated by the WAVE regulatory complex (WRC; Fort *et al.*, 2018 ▸; Whitelaw *et al.*, 2019 ▸). Our structure, the first to be reported of any member of the CYRI family, reveals a protein that is entirely composed of α-helices and organized into three distinct subdomains. CYRI-B exhibits significant structural homology to CYFIP1, a component of the WRC that interacts with the small GTPase Rac1 to promote actin polymerization (Bompard & Caron, 2004 ▸; Pollitt & Insall, 2009 ▸). The similarity in structure suggests that CYRI-B interacts with Rac1 in the same manner as CYFIP1 and therefore regulates actin-dependent processes by competition with the WRC for Rac1 binding. Residues previously described as vital for CYRI-B function (Shang *et al.*, 2018 ▸) cluster on a conserved helix which is likely to be central to Rac1 interaction. This study provides the structural basis to understand the function of CYRI proteins in a variety of fundamental cellular processes.

## Supplementary Material

PDB reference: CYRI-B (FAM49B) from *Rhincodon typus*, 6yjk

PDB reference: selenomethionine derivative, 6yjj

Supplementary Figures and captions to Supplementary Movies. DOI: 10.1107/S2059798320010906/jc5030sup1.pdf

Supplementary Movie S1. DOI: 10.1107/S2059798320010906/jc5030sup2.mp4

Supplementary Movie S2. DOI: 10.1107/S2059798320010906/jc5030sup3.mp4

Supplementary Movie S3. DOI: 10.1107/S2059798320010906/jc5030sup4.mov

## Figures and Tables

**Table 1:** Data-collection and refinement statistics Values in parentheses are for the outer shell.

	SeMet CYRI-B (PDB entry 6yjj)	Native CYRI-B (PDB entry 6yjk)
Data collection		
Temperature (K)	100	100
Wavelength (Å)	0.9611	0.9795
Crystal parameters		
Space group	*P*2_1_2_1_2_1_	*P*2_1_2_1_2_1_
*a*, *b*, *c* (Å)	40.4, 72.8, 107.3	39.8, 72.2, 107.8
α, β, γ (°)	90, 90, 90	90, 90, 90
Reflection data†		
Resolution (Å)	72.82–2.40 (2.49–2.40)	60.01–2.37 (2.46–2.37)
Unique reflections	13006 (1329)	13266 (1362)
*R*_merge_	0.112 (0.965)	0.122 (0.962)
〈*I*/σ(*I*)〉	12.8 (2.1)	10.1 (2.0)
CC_1/2_	0.998 (0.782)	0.998 (0.590)
Completeness (%)	100.0 (100.0)	100.0 (100.0)
Multiplicity	9.5 (8.2)	10.4 (7.5)
Wilson *B* factor (Å^2^)	44.2	47.1
Refinement‡		
Resolution (Å)	53.6–2.40	60.01–2.37
No. of reflections	12959	13207
*R*_work_/*R*_free_	0.2048/0.2415	0.2251/0.2772
R.m.s.d., bond lengths (Å)	0.005	0.006
R.m.s.d., bond angles (°)	0.70	0.84
Model composition		
Protein atoms	2570	2573
Waters	8	8
Other	0	0
Model *B* factors (Å^2^)		
Protein atoms	63.2	74.5
Waters	42.5	45.5
Ramachandran statistics§		
Favoured (%)	97.5	97.5
Allowed (%)	2.5	2.5
Outliers (%)	0.0	0.0

† Reflection data are as reported by *AIMLESS* (Evans & Murshudov, 2013 ▸).

‡ Refinement statistics are as reported by *PHENIX* (Adams *et al.*, 2010 ▸; Liebschner *et al.*, 2019 ▸).

§ Ramachandran statistics are as reported by *RAMPAGE* (Lovell *et al.*, 2003 ▸).
