# Supplementary material for: Structure of CYRI-B (FAM49B), a key regulator of cellular actin assembly
Source: Acta Crystallogr D Struct Biol. 2020 Sep 23;76(Pt 10):1015–24. doi: 10.1107/S2059798320010906 (PMC7543656; doi:10.1107/S2059798320010906)
Supplement: Supplementary file 1 [file d-76-01015-sup1.pdf]

Acta Cryst D STRUCTURAL BIOLOGY

Volume 76 (2020)

Supporting information for article:

Structure of CYRI-B (FAM49B), a key regulator of cellular actin assembly

Elise Kaplan, Rachael Stone, Peter J. Hume, Nicholas P. Greene and Vassilis Koronakis

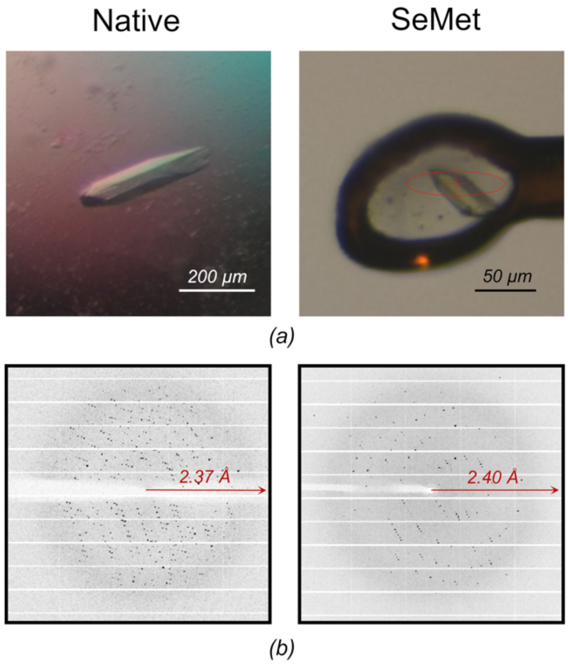

Figure S1 Crystallization and diffraction of *R. typus* CYRI-B (FAM49B). (*a*) Single orthorhombic prism-shaped crystals of native (left) and selenomethionine (SeMet, right) CYRI-B. (*b*) A typical diffraction pattern of native and SeMet CYRI-B (left and right respectively). The resolution limit is indicated.

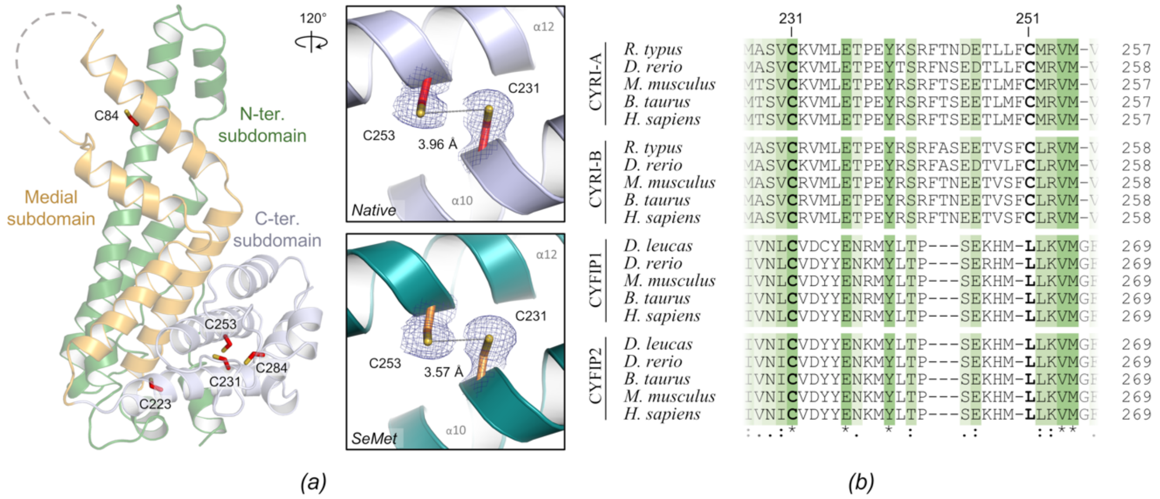

Figure S2 Location of cysteines in CYRI-B, showing a potential C231-C253 disulfide bond. (*a*, left) The five cysteines of CYRI-B are shown as red sticks on a cartoon representation of the *R. typus* crystal structure. (*a*, right) Close-up view showing electron density for the Cys231 and Cys253 side chains on the native (top) and selenomethionine (SeMet) derivative (bottom) proteins, crystallized in the presence of β-mercaptoethanol. The mesh represents a weighted 2|Fo|-|Fc| electron density map contoured at 1 σ. The beta carbons of the two cysteines are 3.7 and 4.1 Å apart in the selenomethionine and native structure, respectively, compatible with the formation of a disulfide bond in non-reducing conditions. (*b*) Multiple sequence alignment of region 227 to 258 (*R. typus* CYRI-B numbering) of CYRI-A, CYRI-B, and 241 to 269 of CYFIP1 and CYFIP2 proteins. Alignment was performed with Clustal Omega (Sievers *et al.*, 2011) and the following sequences: *R. typus*, *Rhincodon typus* (NCBI XP_020388351.1 and XP_020368600.1); *D. rerio*, *Danio rerio* (Q5TZ57, Q6TLE5, Q90YM8 and A5A5E1); *M. musculus*, *Mus musculus* (Q8BHZ0, Q921M7, Q7TMB8 and Q5SQX6); *B. taurus*, *Bos taurus* (Q17QT7, Q2KJI3, E1BN47 and F1MX60); *H. sapiens*, *Homo sapiens* (Q9H0Q0, Q9NUQ9, Q7L576 and Q96F07); *D. leucas*, *Delphinapterus leucas* (A0A2Y9PLS0 and A0A2Y9PRF5). Except otherwise specified, sequences for CYRI-A, CYRI-B, CYFIP1 and CYFIP2 proteins respectively are from Uniprot. Annotation of *R. typus* genome did not allow clear identification of CYFIP proteins, which were substituted by CYFIP1 and CYFIP2 sequences of the related, Beluga whale (*D. leucas*). Residues are coloured according to the conservation symbols given by Clustal Omega.

```
I. furcatus   CYRI-A  MGNLLKVLTREI-ENYPHFFLDFENAQPTECERDVWNQVNAVLQESESILSGLQAYKGAGQEIRD   64
R. typus      CYRI-A  MGNLLKVLTREI-ENYPHFFLDFENAQPTDGEREIWNQVNAVLQDSESILTELQAYKGAGQEIRD   64
G. gallus     CYRI-A  MGNLLKVLTREI-ENYPHFFLDFENAQPTDGEREVWNQISAVLQDSESMLADLQAYKGAGQEIRD   64
X. laevis     CYRI-A  MGNLLKVLTREI-ENYPHFFLDFENAQPTDGEREVWNQVNAVLQDSESILSDLQAYKGAGQEIRD   64
C. horridus   CYRI-A  MGNLLKVLTREI-ENYPHFFLDFENAQPTDGEREIWNQVNAVLQDSESMLSDLQSYKGAGQEIRD   64
H. sapiens    CYRI-A  MGNLLKVLTREI-ENYPHFFLDFENAQPTEGEREIWNQISAVLQDSESILADLQAYKGAGPEIRD   64
F. catus      CYRI-A  MGNLLKVLTREI-ENYPHFFLDFENAQPTEGEREIWNQISAVLQDSESILADLQAYKGAGPEIRD   64
M. musculus   CYRI-A  MGNLLKVLTREI-ENYPHFFLDFENAQPTEGEREIWNQISAVLQDSESILTDLQAYKGAGPEIRD   64
I. furcatus   CYRI-B  MGNLIKVLTRDIDNNAGNFFLDFENAQPTDAERELWEQVNKVLTEAVSVLQDLQAYSGAGESIRQ   65
R. typus      CYRI-B  MGNLIKVLTRDIDHNASHFFLDFENAQPTEAEREIFNQVNVVLKDAEGILNDLQSYRGAGHEIRE   65
G. gallus     CYRI-B  MGNLIKVLTRDIDHNAAHFFLDFENAQPTESEKEIYNQVNVVLKDAEGILEDLQSYRGAGHEIRE   65
X. laevis     CYRI-B  MGNLLKVLTCTDLEQGPNFFLDFENAQPSESEKEVYNQVNVVLKDAEGILDQLQSYRGAGHEIRE   65
C. horridus   CYRI-B  MGNLLKVLTCTDLEQGPNFFLDFENAQPTESEKEIYNQVNVVLKDAEGILEDLQSYRGAGHEIRE   65
H. sapiens    CYRI-B  MGNLLKVLTCTDLEQGPNFFLDFENAQPTESEKEIYNQVNVVLKDAEGILEDLQSYRGAGHEIRE   65
F. catus      CYRI-B  MGNLLKVLTCTDLEQGPNFFLDFENAQPTESEKEIYNQVNVVLKDAEGILEDLQSYRGAGHEIRE   65
M. musculus   CYRI-B  MGNLLKVLTCTDLEQGPNFFLDFENAQPTESEKEIYNQVNVVLKDAEGILEDLQSYRGAGHEIRE   65

        conservation  ****+****564-49665*********897*99997*994**698488*44**8*5***26**7

I. furcatus   CYRI-A  AIQNPNDLLLQERAWTSVCPLVIRLKKFYGFSLKLEKALQSLLESLTCPPYTPTQHLEKEQALAK  129
R. typus      CYRI-A  AIQNPSDGQLQDRAWSSVCPLVGRLKRFYEFSIRLEKALQSLLESLTYPPYTPTQHLEREQALAK  129
G. gallus     CYRI-A  AIQNPNDIQLQEKAWNSVCPLVVRLKRFYEFSLRLEKALQSLLESLTCPPYTPTQHLEREQALAK  129
X. laevis     CYRI-A  AIQNPNDIHLQEKAWNSVCPLVVRLKRFYEFSLRLEKALQSLLESLTFPPYAPTQHLEREQALAK  129
C. horridus   CYRI-A  AIQNPNDIQLQERAWNSVCPLVVRLKRFYEFSLRLEKALQSLLESLTCPPYTPTQHLEREQALAK  129
H. sapiens    CYRI-A  AIQNPNDIQLQEKAWNAVCPLVVRLKRFYEFSIRLEKALQSLLESLTCPPYTPTQHLEREQALAK  129
F. catus      CYRI-A  AIQNPNDIQLQEKAWNAVCPLVVRLKRFYEFSIRLEKALQSLLESLTCPPYTPTQHLEREQALAK  129
M. musculus   CYRI-A  AIQNPNDIQLQEKAWNAVCPLVVRLKRFYEFSIRLEKALQSLLESLTCPPYTPTQHLEREQALAK  129
I. furcatus   CYRI-B  AIQQPSNESVQEKAWTAVVPLVGKLKKFYEFSLKLEGALHGLLGFLTSAHCSPTQHLEQEQALAR  130
R. typus      CYRI-B  AIQHPNDENLQEKAWSAVCPLVGKLKKFYEFSQRLEAALHGLLGALTSTPYSPTQHLEREQALAK  130
G. gallus     CYRI-B  AIQHPNDEKLQEKAWGAVVPLVGKLKKFYEFSQRLEAGLRGLLGALTSTPYSPTQHLEREQALAK  130
X. laevis     CYRI-B  AIQHATDEKLQEKAWAAVVPLVGKLKKFYEFSQRLEAALRGLLGALTSTPYSPTQHLEREQALAK  130
C. horridus   CYRI-B  AIQHPSDERLQEKAWGAVVPLVGKLKKFYEFSQRLEAALRGLLGALTSTPYSPTQHLEREQALAK  130
H. sapiens    CYRI-B  AIQHPADEKLQEKAWGAVVPLVGKLKKFYEFSQRLEAALRGLLGALTSTPYSPTQHLEREQALAK  130
F. catus      CYRI-B  AIQHPADEKLQEKAWGAVVPLVGKLKKFYEFSQRLEAALRGLLGALTSTPYSPTQHLEREQALAK  130
M. musculus   CYRI-B  AIQHPADEKLQEKAWGAVVPLVGKLKKFYEFSQRLEAALRGLLGALTSTPYSPTQHLEREQALAK  130

        conservation  ***5778329*99**78*9***79**9**4**79**5+*68**45**56377******7*****9

I. furcatus   CYRI-A  QFAEILHFTLRFDELKMRIPAIQNDFSYYRRTISRNRINNMNLDIENEVNNEMANRMSLFYAEAT  194
R. typus      CYRI-A  QFAEILHFTLRFDELKMKNPAIQNDFSYYRRTISRNRINNMHLDAENEVNNEMANRMSLFYAEAT  194
G. gallus     CYRI-A  EFAEILHFTLRFDELKMRNPAIQNDFSYYRRTISRNRINNMHLDIENEVNNEMANRMSLFYAEAT  194
X. laevis     CYRI-A  EFAEILHFTLRFDELKMRNPAIQNDFSYYRRTISRNRINNMHLDIENNVNNEMANRMSLFYAEAT  194
C. horridus   CYRI-A  QFAEILHFTLRFDELKMRNPAIQNDFSYYRRTLSRNRINNMHLDIESEVNNEMANRMSLFYAEAT  194
H. sapiens    CYRI-A  EFAEILHFTLRFDELKMRNPAIQNDFSYYRRTISRNRINNMHLDIENEVNNEMANRMSLFYAEAT  194
F. catus      CYRI-A  EFAEILHFTLRFDELKMRNPAIQNDFSYYRRTISRNRINNMHLDIENEVNNEMANRMSLFYAEAT  194
M. musculus   CYRI-A  EFAEILHFTLRFDELKMRNPAIQNDFSYYRRTISRNRINNMHLDIENEVNNEMANRMSLFYAEAT  194
I. furcatus   CYRI-B  QFAEILHFTLRFDELKMTNPAIQNDFSYYRRTLSRMRINNLATEEENEVNNELANRMSLFYANAT  195
R. typus      CYRI-B  QFAEILHFTLRFDELKMTNPAIQNDFSYYRRTLSRMRINNVPAEGENEVNNELANRMSLFYAEAT  195
G. gallus     CYRI-B  QFAEILHFTLRFDELKMTNPAIQNDFSYYRRTLSRMRINNVPAEGENEVNNELANRMSLFYAEAT  195
X. laevis     CYRI-B  QFAEILHFTLRFDELKMTNPAIQNDFSYYRRTLSRMRINNLPAEGENEVNNELANRMSLFYAEAT  195
C. horridus   CYRI-B  QFAEILHFTLRFDELKMTNPAIQNDFSYYRRTLSRMRINNVPAEGENEVNNELANRMSLFYAEAT  195
H. sapiens    CYRI-B  QFAEILHFTLRFDELKMTNPAIQNDFSYYRRTLSRMRINNVPAEGENEVNNELANRMSLFYAEAT  195
F. catus      CYRI-B  QFAEILHFTLRFDELKMTNPAIQNDFSYYRRTLSRMRINNVPAEGENEVNNELANRMSLFYAEAT  195
M. musculus   CYRI-B  QFAEILHFTLRFDELKMTNPAIQNDFSYYRRTLSRMRINNVPAEGENEVNNELANRMSLFYAEAT  195

        conservation  8****************66************+**7****82693*97****9*********7**

I. furcatus   CYRI-A  PMLKTLSTATTNFVTENKTLPLENTTDCLSTMASVCKVMLETPEYTSRFNSEDTLLFCMRVMVGV  259
R. typus      CYRI-A  PMLKTLSDATTKFVSENKTLPIENTTDCLSTMASVCKVMLETPEYKSRFTNDETLLFCMRVMVGV  259
G. gallus     CYRI-A  PMLKTLSNATTHFVSENKTLPIENTTDCLSTMASVCKVMLETPEYRSRFTSEETLMFCMRVMVGV  259
X. laevis     CYRI-A  PMLKTLSNATTSFVSDNKTLPIENTTDCLSTMASVCKVMLETPEYSSRFTSEDTLMFCMRVMVGV  259
C. horridus   CYRI-A  PVLKTLSNATTHFVVENKTLPIENTTDCLSTMASVCKVMLETPEYRSRFTSEETLMFCMRVMVGV  259
H. sapiens    CYRI-A  PMLKTLSNATMHFVSENKTLPIENTTDCLSTMTSVCKVMLETPEYRSRFTSEETLMFCMRVMVGV  259
F. catus      CYRI-A  PMLKTLSNATMHFVSENKTLPIENTTDCLSTMTSVCKVMLETPEYRSRFTSEETLMFCMRVMVGV  259
M. musculus   CYRI-A  PMLKTLSNATMHFVSENKTLPIENTTDCLSTMTSVCKVMLETPEYRSRFTSEETLMFCMRVMVGV  259
I. furcatus   CYRI-B  PMLKTLSDATTKFVSENSDVPIENTTDCLSTMACVCKVMLDTPEYRSRFASEDTVLFCLRVMVGV  260
R. typus      CYRI-B  PMLKTLSDATTKFVSDNKSLPIENTTDCLSTMASVCRVMLETPEYRSRFASEETVSFCLRVMVGV  260
G. gallus     CYRI-B  PMLKTLSDATTKFVSENKNLPIENTTDCLSTMASVCRVMLETPEYRSRFTNEETVSFCLRVMVGV  260
X. laevis     CYRI-B  PMLKTLSDATTKFVSENKNLPIENTTDCLSTMASVCRVMLETPEYRSRFTNEETVSFCLRVMVGV  260
C. horridus   CYRI-B  PMLKTLSDATTKFVSENKNLPIENTTDCLSTMASVCRVMLETPEYRSRFTNEETVSFCLRVMVGV  260
H. sapiens    CYRI-B  PMLKTLSDATTKFVSENKNLPIENTTDCLSTMASVCRVMLETPEYRSRFTNEETVSFCLRVMVGV  260
F. catus      CYRI-B  PMLKTLSDATTKFVSENKNLPIENTTDCLSTMASVCRVMLETPEYRSRFTNEETVSFCLRVMVGV  260
M. musculus   CYRI-B  PMLKTLSDATTKFVSENKNLPIENTTDCLSTMASVCRVMLETPEYRSRFTNEETVSFCLRVMVGV  260

        conservation  *8*****7**84**69*569*+**********87**9***9****5***7999*95**9******
```

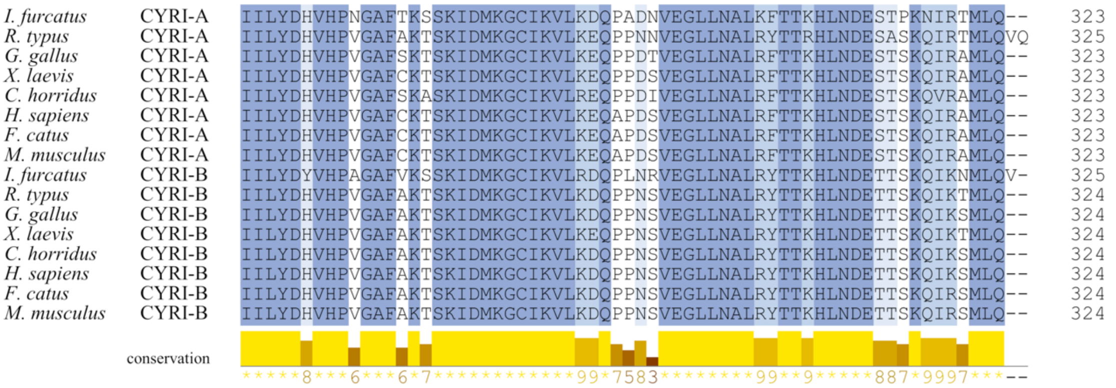

(a)

CYRI-A (rows) × CYRI-A (columns)

	I. furcatus	*R. typus*	*G. gallus*	*X. laevis*	*C. horridus*	*H. sapiens*	*F. catus*	*M. musculus*
I. furcatus	100							
R. typus	86	100						
G. gallus	88	91	100					
X. laevis	88	90	96	100				
C. horridus	87	90	95	93	100			
H. sapiens	86	90	97	94	93	100		
F. catus	86	90	97	94	93	100	100	
M. musculus	86	90	96	94	93	100	100	100

CYRI-B (rows) × CYRI-A (columns)

	I. furcatus	*R. typus*	*G. gallus*	*X. laevis*	*C. horridus*	*H. sapiens*	*F. catus*	*M. musculus*
I. furcatus	74	75	74	74	74	73	73	73
R. typus	77	81	80	81	80	81	81	81
G. gallus	76	81	79	79	79	80	80	80
X. laevis	75	80	79	79	78	79	79	79
C. horridus	75	81	79	79	79	80	80	80
H. sapiens	75	81	79	79	79	80	80	80
F. catus	76	81	79	79	79	80	80	80
M. musculus	76	81	79	79	79	80	80	80

CYRI-B (rows) × CYRI-B (columns)

	I. furcatus	*R. typus*	*G. gallus*	*X. laevis*	*C. horridus*	*H. sapiens*	*F. catus*	*M. musculus*
I. furcatus	100							
R. typus	82	100						
G. gallus	81	95	100					
X. laevis	79	91	94	100				
C. horridus	80	93	96	97	100			
H. sapiens	80	93	96	97	99	100		
F. catus	79	92	96	97	99	100	100	
M. musculus	79	92	96	97	99	100	100	100

(b)

Figure S3 Sequence alignment of CYRI (FAM49) proteins across different species. (*a*) Alignment was generated with Clustal Omega (Sievers *et al.*, 2011) using the following protein sequences: *I. furcatus*, *Ictalurus furcatus* (Uniprot E3TCC8 and E3TCX7); *R. typus*, *Rhincodon typus* (NCBI XP_020388351.1 and XP_020368600.1); *G. gallus*, *Gallus gallus* (Uniprot Q5ZI04 and A0A3Q2TTC4); *X. laevis*, *Xenopus laevis* (Uniprot Q0IHF5 and Q8AVE9); *C. horridus*, *Crotalus horridus* (Uniprot A0A0K8RVD5 and A0A0B8RNP7); *H. sapiens*, *Homo sapiens* (Uniprot Q9H0Q0 and Q9NUQ9); *F. catus*, *Felis catus* (Uniprot A0A5F5XX33 and M3WMY8); *M. musculus*, *Mus musculus* (Uniprot Q8BHZ0 and Q921M7). Conservation at each (0, least conserved; 9 most conserved) was assessed in Jalview 1.0 and displayed beneath the sequence alignment. (*b*) Identity matrix based on the aligned sequences, generated by Clustal Omega.

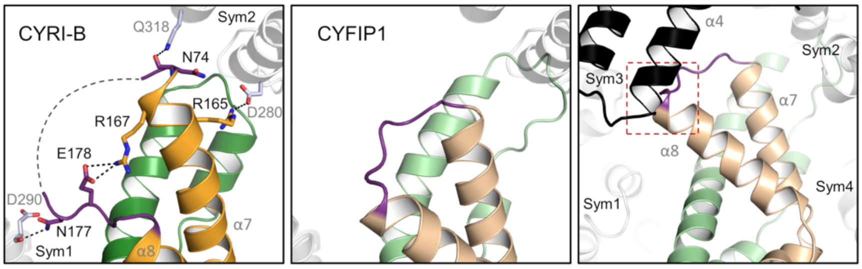

(a) (b) (c)

Figure S4 Close-up view of the disordered loop in *R. typus* CYRI-B. The loop that connects helices α7 and α8 is shown in purple in our crystal structure of CYRI-B (*a*) or in CYFIP1 (*b*, PDB 3P8C). It is composed of residues 169 to 181 in CYRI-B (195-201 in CYFIP1) and residues 171-175 were too disordered to model. Main local interactions between residues of the Medial subdomain and residues belonging to symmetry mate proteins are represented in CYRI-B structure. (*c*) Close-up view of CYFIP1 aligned on CYRI-B structure showing steric clash with one of the CYRI-B symmetry mates (black). A dotted red square highlights the position of the steric hindrance.

Movie S1 Representative electron density of the native CYRI-B (FAM49B) structure. A weighted 2|Fo|-|Fc| electron density map, calculated with model phases, is shown as grey mesh contoured at 1 σ.

Movie S2 Camera tour of the selenomethionine (SeMet) derivatized CYRI-B (FAM49B) structure. The first half of the movie shows the anomalous difference Fourier map surrounding selenium atoms as a blue mesh contoured at 3 σ. The second half of the film shows a weighted 2|Fo|-|Fc| electron density map, calculated with model phases, as grey mesh contoured at 1 σ.

Movie S3 Electrostatic surface of whale shark and human CYRI-B. Full 360º rotation movie around the y-axis showing the electrostatic surface of the whale shark CYRI-B crystal structure, left, and human homology model, right.
